# Efficient Electrochemical Methods for Low-Loading Ru Deposition on Carbon Electrodes as a Hydrogen Evolution Reaction Catalyst in an Acidic Environment

**DOI:** 10.1021/acsaem.4c03349

**Published:** 2025-04-29

**Authors:** Rachela Gabriella Milazzo, Nino Marino, Giuseppe Tranchida, Corrado Bongiorno, Luca Pulvirenti, Letizia Fusto, Guglielmo Guido Condorelli, Salvatore Antonino Lombardo, Stefania Maria Serena Privitera

**Affiliations:** † CNR-IMM VIII Strada 5, Catania 95121, Italy; ‡ Department of Chemical Sciences, University of Catania, Viale Andrea Doria, 6, Catania 95125, Italy

**Keywords:** green hydrogen, sustainable catalysts, electrodeposition, water splitting, ultra low loading

## Abstract

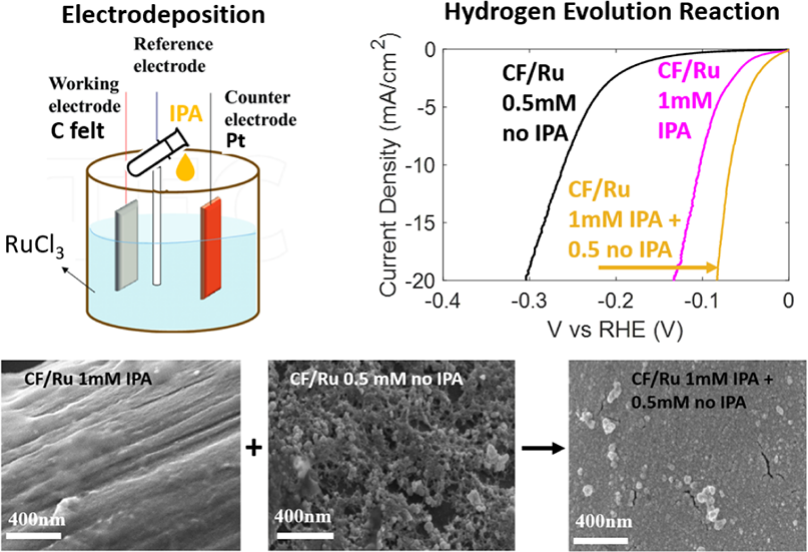

The widespread development of technologies for green hydrogen production strictly relies on the availability of durable electrocatalysts that can operate in either acidic or alkaline electrolytes while using a limited amount of platinum group metals. In this work, we present an effective strategy based on electrodeposition as a low-cost method to obtain low-loading Ru catalysts on carbon electrodes for the hydrogen evolution reaction in an acidic environment. The deposition conditions have been investigated and optimized in order to have uniform coverage, a large number of active sites, and good electrocatalytic performance. The morphology and chemical structure have been investigated using scanning electron microscopy and X-ray photoelectron spectroscopy measurements. Excellent catalytic activity has been achieved with a Ru loading of 0.06 mg cm^–2^, obtaining an overpotential of 67 mV at 10 mA cm^–2^ and a Tafel slope of 50 mV dec^–1^.

## 1 Introduction

Hydrogen has the potential to play a significant role in addressing global warming impacts and reducing greenhouse gas emissions. In particular, green hydrogen production through water electrolysis using the surplus electric power produced by renewable energy sources relies directly on the development of efficient electrocatalysts for the hydrogen evolution reaction (HER). While the HER can be performed in either alkaline or acidic environments, the latter condition is faster and easier owing to the high concentration of protons, although it is limited by the corrosion of catalysts. In particular, proton exchange membrane (PEM) electrolysis is a technology capable of producing high-purity hydrogen gas at much higher current densities compared with alkaline electrolysis. Due to their outstanding catalytic activity and chemical stability in harsh acidic and oxidizing conditions, platinum group catalysts are commonly adopted as catalysts in PEM electrolysis. Platinum (Pt) is the most effective HER catalyst in acid electrolytes, and it is commonly employed with typical loadings between 0.5 and 1.0 mg_Pt_ cm^–2^.^1^ In commercial applications, loading is usually obtained by catalyst coating of the membrane (CCM) with inks. However, considering the high cost of Pt and its scarcity, for large-scale deployment of PEM systems, it is necessary to reduce its use. While research on lower catalyst loadings based on CCM mainly focuses on ink composition, membrane coating techniques, carbon support, and ionomer content,^2^ other alternative solutions have been proposed, such as alloying Pt with other non-noble transition metals, fabricating it on earth-abundant substrates, or by forming structures with large surface-to-volume ratios.^3−5^ A promising approach is the use of porous transport electrodes (PTE) directly coated with the catalyst.^6^ Such an approach has the advantage of adopting a sustainable material like carbon as a porous support and covering the whole surface of the porous layer, thereby improving the active surface area. Compared with CCM cells, catalyst-coated PTE has shown comparable cell performance at low current densities and improved performance at higher current densities.^7^ Moreover, it can be relevant to reduce production costs through large-volume production.^8^ PTE catalyst coating by magnetron sputtering^9^ or atomic layer deposition (ALD)^10−12^ has recently been demonstrated as a promising method for catalyst loading reduction.

In this paper, we propose electrodeposition as an efficient, facile, and versatile method to produce carbon-based PTE with a low loading of Ruthenium (Ru) catalyst, to be adopted in an acidic environment. The main advantages of electrodeposition are its suitability to cover even the internal surfaces of porous electrodes^13^ and the possibility of obtaining highly porous catalyst layers, with an increased surface-to-volume ratio.^14,15^ Ruthenium represents an interesting alternative to Pt since it shares similar intrinsic activity with it in hydrogen bond strength (≈65 kcal mol^–1^ for Ru and ≈62 kcal mol^–1^ for Pt)^16^ and possesses the merits of relatively low cost (∼20% of the cost of Pt) and excellent durability, thereby becoming an ideal alternative to Pt for the HER. Recent advances in Ru-based HER catalysts have mainly focused on the synthesis of dispersed nanoparticles or even single-atom catalysts^17^ supported on different substrates^18,19^ such as Ru/CeO_2_,^20^ Ru/MoS_2_,^21^ Ru alloy,^22^ and Ru/graphene.^23^ However, the processes for the preparation of the catalysts mentioned above are usually complicated: they require a large number of steps or special facilities to deal with harsh reaction conditions.^24,25^ Recently, the excellent stability of Ru on Ni foam obtained by a wet chemical approach has been demonstrated, especially in an alkaline environment,^26^ obtaining a low overpotential of 22 mV to support a current density of 10 mA cm^–2^ with a Tafel slope of 33 mV dec^–1^ in KOH electrolyte. Although Ru on Ni foam has been found to perform quite well in acidic electrolyte as well, it exhibits less satisfactory performance and stability, mainly because Ni electrodes are not stable in acid and may be easily corroded.^27^ Therefore, a facile synthesis of a Ru layer with a low amount of catalyst on a supporting electrode suitable for long-term operation in an acidic environment is worth pursuing. With this aim, in order to ensure efficient channels for the diffusion of protons and the transit of the bubbles inside the electrode, carbon felt (CF) with hydrophobic treatment and microporous layer has been chosen as the porous support with high electrical conductivity, excellent chemical stability, and mechanical flexibility. The HER activity is related to the number of exposed active sites of catalysts; therefore, the ability to fabricate Ru-based nanostructures with tunable porosity and active sites is considered a decisive point for the catalyst design. In this paper, different Ru electrodeposition conditions have been investigated, including the addition of isopropyl alcohol in the deposition solution, in order to optimize the coverage and morphology required to achieve the best electrochemical performance in terms of active surface area, overpotential, and Tafel slope. The optimized catalyst shows excellent catalytic activity toward the HER in H_2_SO_4_, while maintaining a Ru loading amounting to 0.06 mg cm^–2^, well below the limit of 0.5 mg cm^–2^ recommended by the U.S. Department of Energy Fuel Cell Technologies Program^28^ for both electrodes combined.

## 2 Experimental Methods

### 2.1 Fabrication of the Electrodes: Ruthenium Electrodeposition on C-Felt

Ruthenium-loaded electrodes were prepared by electrodeposition on C-felt substrates according to the following procedure: C-felt (CF) H23C9, provided by Freudenberg, was adopted as a substrate. The CF has a thickness of 250 μm and is typically adopted as a gas diffusion electrode, being prepared by the supplier with a single-sided microporous layer coated with a hydrophobic layer. The electrical resistance of the C-felt substrate is 0.7 Ohm (in-plane) and the specific area, calculated using the BET (Brunauer–Emmett–Teller) theory, has been reported to be 0.37 m^2^g^–1^,^28^ corresponding to a surface area of 2000 cm^2^ cm^–3^. The CF pieces (1 cm × 1 cm) were immersed in HCl solution (10 mM) with 0.5 or 1 mM RuCl_3_. The electrodepositions were performed at a current density of 1.5 mA cm^–2^ in a two-electrode cell with a Pt counter electrode. The solution was continuously stirred at 300 rpm during the electrodeposition, and for some samples, isopropyl alcohol (IPA) was added. After deposition optimization, one sample was deposited in double steps: for step 1, we adopted a solution containing 1 mM RuCl_3_, 10 mM HCl, 5% vol. IPA; for step 2, the RuCl_3_ concentration was lowered to 0.5 mM, and IPA was not added to the bath.

As a reference, Pt was electrodeposited on C-felt electrodes^29^ in a solution of 1 mM Na_2_PtCl_6_ and 10 mM HCl for 1000 s while maintaining a current density of 1.5 mA cm^–2^. After electrodeposition, all the electrodes were rinsed in deionized water and dried in a nitrogen atmosphere.

### 2.2 Morphological and Electrochemical Characterization

For the morphological, structural, and chemical analyses, we adopted a scanning electron microscope (SEM) Helios 5 Dual Beam by Thermo Fisher. The SEM was employed to perform energy-dispersive X-ray (EDS) spectroscopy, thus identifying the chemical elements present in different regions of the electrodes.

The chemical state of the deposited ruthenium was investigated by X-ray photoelectron spectroscopy (XPS). Spectra were acquired with a PHI 5600 multitechnique ESCA-Auger spectrometer equipped with a standard Mg Kα X-ray source. Samples were analyzed with a photoelectron takeoff angle of 45° (relative to the sample surface) and with an acceptance angle of ± 7°. The XPS binding energy scale was referenced by centering the C 1s peak at 285.0 eV.

For the electrochemical characterization, linear wweep voltammetry (LSV), chronopotentiometry (CP), and electrochemical Impedance Spectroscopy (EIS) were performed using a potentiostat/galvanostat VERTEX by Ivium Tech. The electrochemical data were compensated for the ohmic drop potential (*iR*), and the impedance at open circuit was analyzed over a frequency range from 10 kHz to 0.1 Hz. All measurements were performed at room temperature in a three-electrode configuration, adopting a Pt wire as the counter electrode. The HER activity was evaluated in 0.1 M H_2_SO_4_ electrolyte, with Ag/AgCl (3 M KCl) as the reference electrode. The measured potentials E were converted to the potential vs RHE (E_RHE_) using the Nerst eq 1, which is as follows:
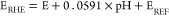
1

where E_REF_ is the potential of the reference electrode vs RHE (0.207 V for Ag/AgCl). The stability was investigated by monitoring the voltage as a function of time (transient chronopotentiometry, TRCP) during a fixed current density of −5 mA cm^–2^.

Additionally, to provide a comparison with the acidic medium, the activity of the Ru-loaded CF electrode prepared by double-step deposition has also been tested in a neutral electrolyte (0.1 M Na_2_SO_4_). The electrode performance in a neutral medium has been tested by performing chronopotentiometry at −5 mA cm^–2^ and −10 mA cm^–2^.

### 2.3 Loading Evaluation

It is known from the literature^30^ that RuCl_3_ has an adsorption peak around 535 nm. It is therefore possible to determine the amount of Ru in a solution by UV–vis spectrometry. We have adopted a spectrometer (Cary 5000 UV–vis-NIR, Agilent) to measure the absorbance spectra of a set of RuCl_3_ solutions with well-known concentrations to obtain a calibration curve (Figure SI 1a,b). After the calibration procedure, we acquired the absorbance spectra of the RuCl_3_ solutions employed for electrodeposition before and after Ru deposition on C-felt substrates. The Ru loading was calculated as the difference between the Ru amount in the starting solution and that measured after electrodeposition (Figure SI 1c). We adopted a spectrophotometric method for the evaluation of Ru loading because it has the advantage of being a nondestructive technique and allows measuring the loading exactly in the same electrode adopted for the electrochemical evaluation.

## 3 Results and Discussion

### 3.1 Ru Electrodeposition

Ru electrodeposition on a C-felt has been conducted at the cathode of a two-electrode cell at a constant current of −1.5 mA cm^–2^ in order to produce as many metal crystal seeds as possible and to avoid Ru oxidation. This current corresponds to a cell voltage sufficient to produce the hydrogen evolution reaction at the cathode, in competition with Ru deposition. As a consequence, hydrogen bubbles may form and grow in size, reducing the efficiency of deposition. To mitigate this effect, the solution was continuously stirred, and in some cases, IPA was added to the solution. The chronopotentiometry acquired during Ru deposition with and without IPA is shown in Figure SI 2. IPA is an organic polar molecule that is usually adopted in industrial processes to promote wettability. Among other applications, it is used in the offset printing industry to lower the surface tension on porous paper^31^ as well as in the microelectronics and photovoltaic industries. Many papers have reported that IPA increases the wettability of the silicon surface during anisotropic etching in alkaline solutions (NaOH or KOH) and facilitates the removal of adhering hydrogen bubbles sticking to the Si surface.^32,33^ Other effects of IPA reported in the literature include the reduction of the etching rate of the silicon wafer^34,35^ and the improvement of surface morphology.^36^

**Figure 1:**
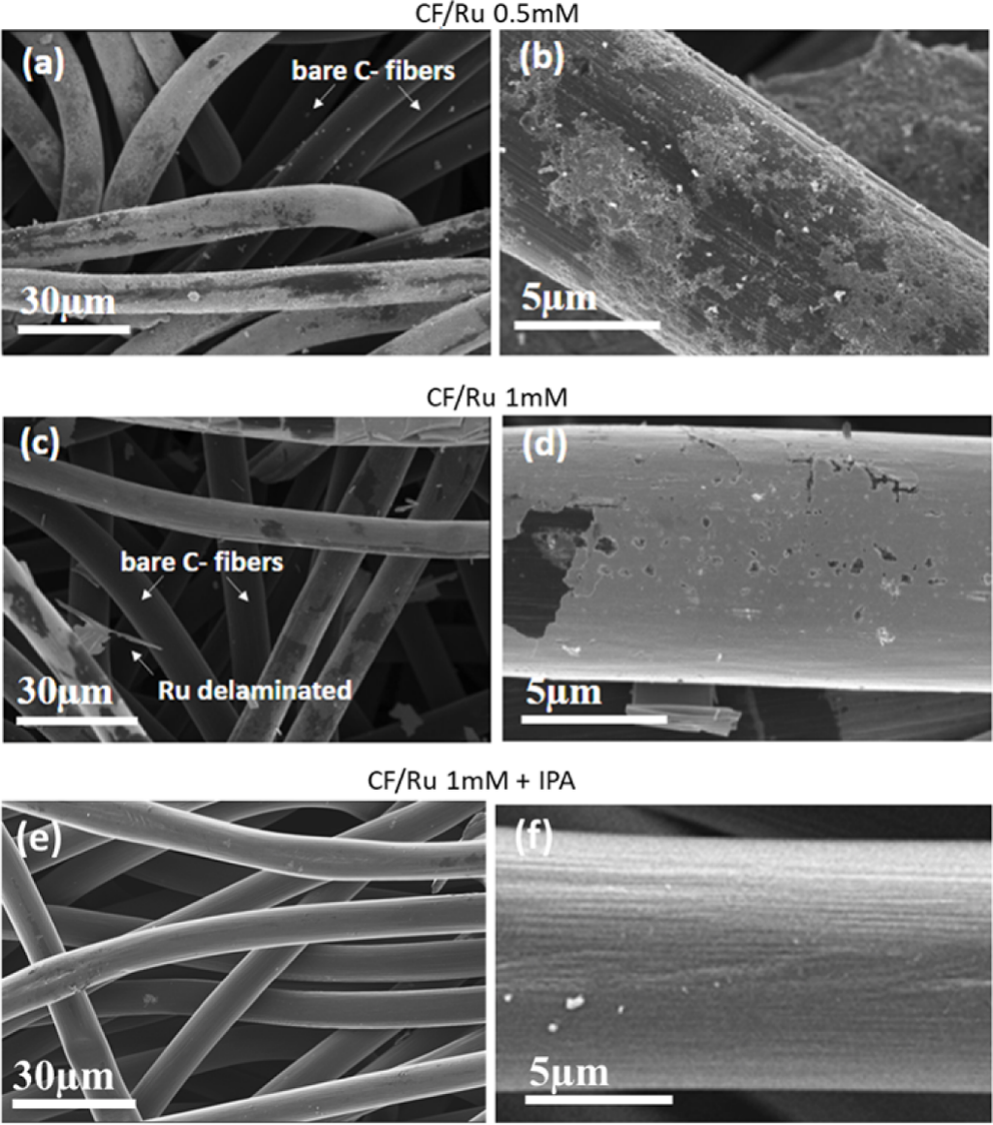
SEM micrographs of C-felt electrodeposited in a solution containing 10mM of HCl and RuCl_3_ with concentrations of 0.5 mM (a–b) or 1 mM without (c–d) and with (e–f) IPA addition.

Ru-loaded C-felt electrodes, prepared according to different electrodeposition conditions, have been investigated using SEM, in order to study the effects of RuCl_3_ concentration and IPA addition on their morphology . Figure 1 shows the micrographs of C-felt after deposition in a 0.5 mM RuCl_3_ aqueous solution (Figure 1 a,b) or 1 mM (Figure 1 c,d). Using 0.5 mM RuCl_3_, the Ru coverage is not uniform and tends to form islands rather than a film. By increasing the Ru concentration in the deposition bath up to 1 mM, although in some fibers, a more continuous layer can be observed, it appears to lack mechanical stability , exhibiting crack formation and delamination. This leads to some uncovered areas in the carbon felt, as shown in Figure 1c,d. Moreover, Ru is not deposited on the inner fibers. Images at a higher magnification are shown in Figure SI 3.

The C-felt covered by using 1 mM RuCl_3_ with the addition of IPA is shown in Figure 1 e,f. In this case, a continuous film is obtained, with almost complete coverage of the fibers, and the deposition occurs even in the inner parts. In Figure SI 4 we show the EDS analysis of the electrode CF/Ru 1 mM with IPA. The acquisitions made on different areas of the sample demonstrated that Ru is present everywhere in the form of a very thin and smooth layer (see Table SI 1 for the atomic concentration).

**Figure 2:**
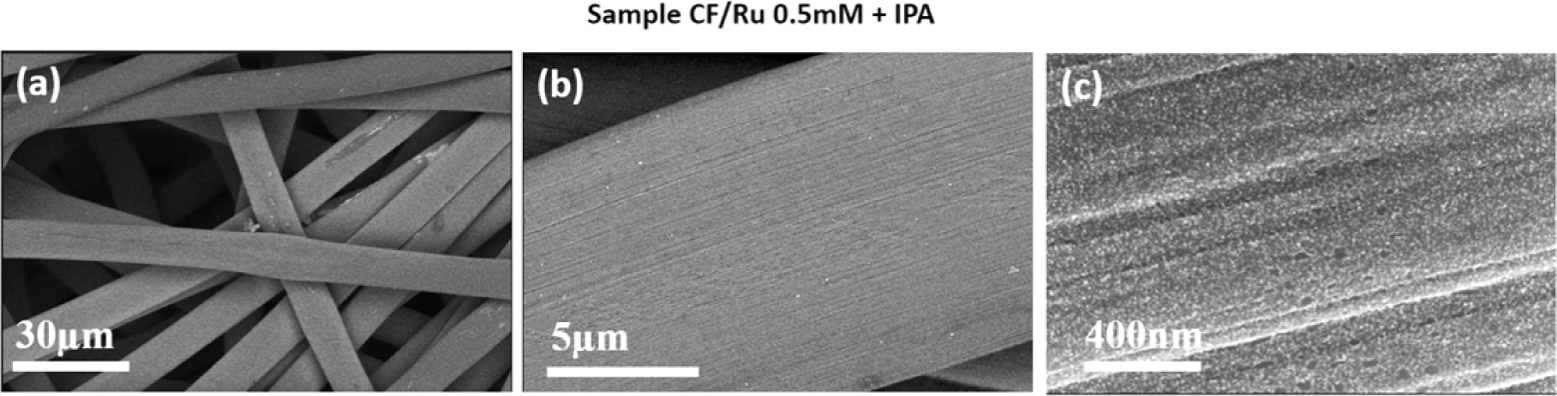
SEM micrographs of C-felt electrodeposited with RuCl_3_ 0.5 mM with IPA at a low (a), medium (b), and higher magnification (c).

Figure 2 shows the micrographs of C-felt after deposition in 0.5 mM RuCl_3_ solution with IPA addition (4% vol). It is clear that the addition of IPA largely affects the morphology of the deposited layer, promoting the deposition of a more uniform, smooth layer, which also reaches the internal fibers of the carbon felt (Figure 2a). Images at a higher magnification (Figure 2c) show that the deposited Ru forms a smooth, almost continuous but porous film, with some small holes, suggesting that the carbon fibers are not completely covered.

**Figure 3:**
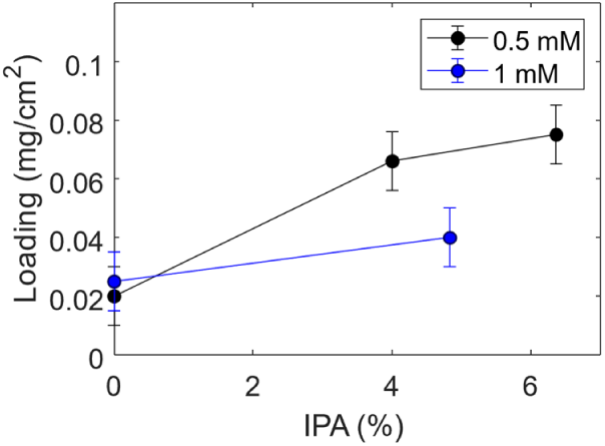
Ru loading after deposition with different deposition solutions (0.5 or 1 mM) as a function of IPA vol. concentration.

In order to better study the effect of surfactant addition, the volume of IPA in the deposition solution was varied, and the corresponding Ru loading was determined by UV–vis spectroscopy. Figure 3 shows the Ru loading after electrodeposition on a C-felt as a function of IPA concentration.

The addition of IPA during electrodeposition influences the Ru nucleation and growth process through several aspects, including the wettability of the carbon substrate, ion transport, and reduction of hydrogen evolution, ensuring a uniform deposition. More specifically, first of all, IPA reduces the surface tension of the electrolyte, ensuring better wetting of the porous carbon felt structure. This behavior is commonly observed in metal electrodeposition when surfactants or organic additives are introduced.^37^ Such an effect leads to a more uniform Ru ion distribution across the electrode surface, promoting homogeneous nucleation even in the inner fibers of the carbon felt and thereby enabling a more uniform coverage of the substrate (Figures 1 e,f and 2). Second, the addition of IPA modifies the local solvation environment of Ru ions, slowing down their diffusion and reduction kinetics. This modifies the morphology of the deposited layer, facilitating the formation of smaller, more uniformly distributed nuclei instead of irregular or agglomerated deposits. Furthermore, IPA, adsorbing on the electrode surface, can inhibit the competing hydrogen evolution reaction occurring during Ru electrodeposition, preventing hydrogen bubble formation that disrupts uniform deposition.^38^ This results in a more efficient electrodeposition process, as confirmed by the increased amount of deposited Ru when IPA is added to the solution, as shown in Figure 3, and by the reduction of cell voltage during electrodeposition (Figure SI 2).

Some electrodes were prepared through a two-step process. For step 1, 5 vol % IPA was added to a 1 mM RuCl_3_ solution and Ru was electrodeposited for 1000 s. The electrode was removed from the deposition bath and rinsed. For step 2, the same electrode was immersed in a fresh 0.5 mM RuCl_3_ solution without IPA, and the electrodeposition continued at the same current (1.5 mA cm^–1^) for other 1000s (sample CF/Ru double). The purpose of this process was to obtain a uniform coverage of all of the fibers during step 1, and to enhance the surface roughness during step 2, in order to increase the active surface area. For comparison, another electrode was prepared by doubling the deposition time, thus obtaining 2000 s deposition with 1 mM RuCl_3_ solution with IPA. The comparison among the LSV, shown in Figure SI 5, demonstrates that the electrochemical performance of the two electrodes, CF/Ru 1 mM IPA 2000 s and CF/Ru double (1 mM IPA + 0.5 mM no IPA), is markedly different, and much better performance can be achieved with the double-step deposition.

**Figure 4:**
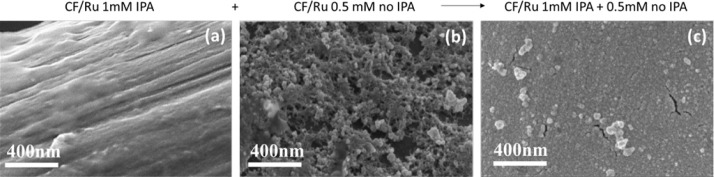
SEM micrographs at a high magnification showing the surface of (a) C-felt electrodeposited with 1mM RuCl_3_ with IPA; (b) C-felt electrodeposited with 0.5mM RuCl_3_ without IPA addition; and (c) a two-step deposition including the first deposition in 1 mM RuCl_3_ with IPA and then in 0.5 mM without IPA.

The effectiveness of the proposed approach in terms of morphology optimization was confirmed by SEM analyses. Figure 4 shows the comparison between (a) the electrode prepared in one step with 1 mM RuCl_3_, with IPA, resulting in a very smooth continuous film; (b) the electrode prepared in one step with 0.5 mM RuCl_3_, without IPA, exhibiting a rough, very porous, but nonuniform layer; and (c) the electrode prepared in two steps (CF/Ru double), with a uniform and continuous coverage and also a higher surface-to-volume ratio.

### 3.2 Electrochemical Performance for the HER

**Figure 5:**
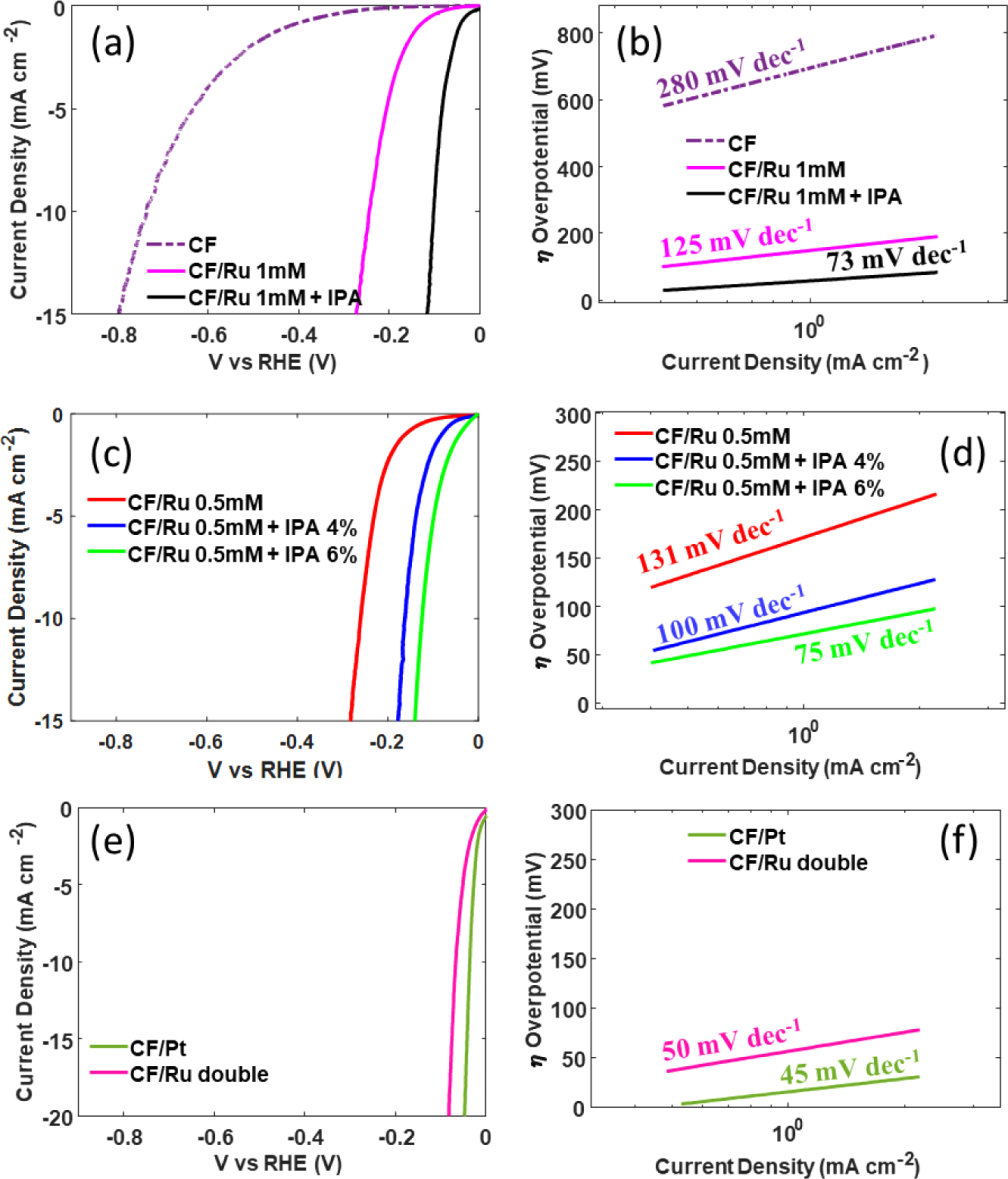
Electrochemical analyses of CF/Ru electrodes. (a), (c), and (e) LSV acquired with a scan rate of 0.5 mV s^–1^ for Ru electrodeposited in 1 mM RuCl_3_, 0.5 mM RuCl_3_, or with the double deposition, respectively. (b), (d), and (f) The corresponding calculated Tafel slopes.

The comparison between the electrochemical performance of various Ru electrodes is shown in Figure 5 together with those of bare CF and CF/Pt for comparison. Figure 5a and c shows the LSV (*iR*-corrected) curves of Ru-loaded electrodes, evidencing the effect of the addition of IPA in the deposition solution containing 1 mM or 0.5 mM RuCl_3_, respectively. In all cases, the addition of IPA gives rise to better performance in terms of overpotential.

The computed Tafel slopes are reported in Figure 5b and d for 1 mM and 0.5 mM RuCl_3_, respectively. The Tafel slope is the number of mV required to increase the current by a factor of 10 (so it is given in mV dec^–1^). Under certain conditions, including no other processes contributing to the current, no mass transport limitations, and no ohmic losses, the rate-determining step can be extracted from the value of the Tafel slope.

It is widely accepted that the formation of hydrogen at the cathode occurs via a multistep electrochemical process.^39,40^ In acidic conditions, the multistep process includes the following three reactions:^41^
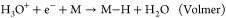
2
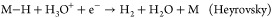
3
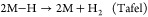
4

The first step involves hydrogen adsorption onto the electrode-active site M (Volmer step), with the formation of hydrogen intermediates (M–H). The subsequent formation of H_2_ involves the hydrogen evolution reaction of an adsorbed atom with another H^+^ (Heyrovsky step) and the H_2_ generation through chemical desorption of two adsorbed hydrogen atoms (Tafel step). As widely accepted, Tafel slopes of 120, 40, and 30 mV dec^–1^ have been observed for the Volmer, Heyrovsky, and Tafel rate-determining steps, respectively.^42^ However, in multistep mechanisms where the surface coverage of reaction intermediates may change, the kinetic analysis can be more complicated, giving rise to ambiguities in the determination of the rate-limiting step. Despite the detailed kinetics mechanism, the Tafel slope is a valuable indicator of the activity of a catalyst, with low values associated with active catalysts, as a smaller overpotential is required to reach a higher current density. In the case of bare C-felt, the measured Tafel slope is 280 mV dec^–1^, typical of C-based electrodes and attributed to its poor conductivity, which also enables a low diffusion rate to the surface electrode. The values obtained for Ru 0.5 mM or Ru 1 mM with no surfactant addition are 131 and 125 mV dec^–1^, respectively, indicating that in this case, the HER kinetics is still limited by H^+^ adsorption on the active sites, according to a Volmer–Heyrovsky mechanism. This is reasonable due to the incomplete coverage of the carbon felt with Ru (see Figure 1 b and d). The addition of IPA results in lower Tafel slopes, equal to 100 mV dec^–1^ and 75 mV dec^–1^ for Ru 0.5 mM with IPA 4% or 6%, respectively, and 73 mV dec^–1^ for Ru 1 mM with IPA 5%, suggesting that the HER process reasonably occurs through a mixed Heyrovsky–Tafel mechanism.

The LSV curve acquired for the CF/Ru double electrode is shown in Figure 5e and compared to that of Pt on CF. The measured overpotential at 10 mA cm^–2^ is 67 mV, close to that of Pt (55 mV). The Tafel slope, evaluated in Figure 5f, is also quite similar to that obtained for the CF/Pt electrode.

**Figure 6:**
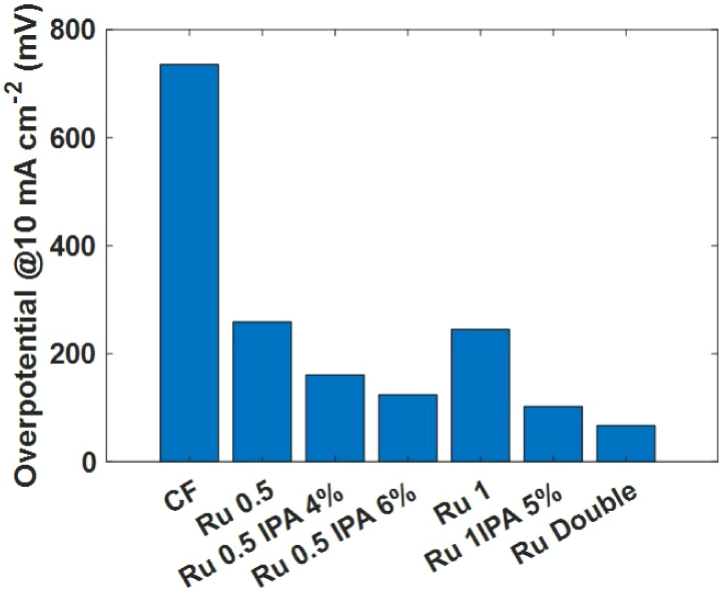
Overpotential measured at 10 mA cm^–2^ in all of the prepared electrodes.

The measured overpotentials η for all the studied electrodes are summarized in Figure 6, and the data are reported in Table 1.

**Table 1:** Electrochemical Data of the CF/Ru Electrodes

	η (mV) @10 mA cm^–2^	Tafel _slope_ (mV dec^–1^)
CF/Ru 0.5	260	131
CF/Ru 0.5 IPA 4%	160	100
CF/Ru 0.5 IPA 6%	124	75
CF/Ru 1	245	125
CF/Ru 1 IPA 5%	103	73
DOUBLE	67	50
CF	753	280

**Figure 7:**
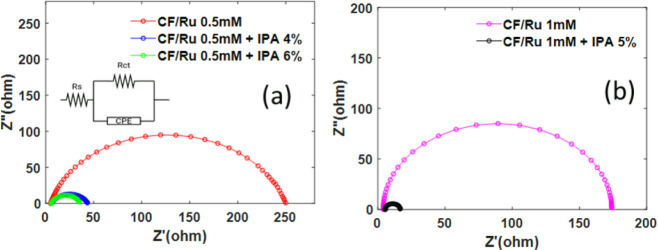
Electrochemical impedance spectroscopy (EIS) spectra (Nyquist plots) and the equivalent circuit adopted to compute Rs, R_ct_, Y_dL_, and α parameters, for the electrodes deposited in 0.5 mM RuCl_3_ (a) or 1 mM RuCl_3_ (b).

Although the Tafel analysis offers a useful approach to determine the reaction rate, it may be influenced by other factors, such as the catalyst area or hydrogen coverage. Therefore, as a complementary method, we also adopted electrochemical impedance spectroscopy (EIS) to fully characterize the catalyst. The EIS measurement involves applying a sinusoidal potential perturbation to the working electrode at different frequencies and measuring the resulting current response. The obtained data are illustrated through the Nyquist plot, which is a common way of representing and analyzing EIS results. The Nyquist plot, obtained by plotting the imaginary part of the impedance (usually -Z″) as a function of the real part of the impedance (Z′) is shown in Figure 7. For all the studied electrodes, the plot has been obtained in the frequency range from 10 kHz to 0.1 Hz. At high frequency, the impedance is dominated by the solution resistance R_s_, while at lower frequency, it is determined by the interaction between the solution and the catalysts through the charge transfer resistance (R_ct_) and double-layer capacitance (C_dL_). In order to evaluate R_ct_ and C_dL_, from the impedance data, a simple equivalent circuit containing solution resistance in series with a constant phase element (CPE) has been adopted, as shown in the inset of Figure 7 a.

The average double-layer capacitance, C_dL_ (in F), can be evaluated using eq 5, as reported in ref.^43^
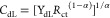
5where *R*_ct_ is the charge transfer resistance, *Y*_dL_ is the capacitance parameter in F s ^α – 1^, and α is the CPE exponent. The results of the fitting are reported in Table 2.

**Table 2:** Data Extracted from EIS Measurements and the Corresponding Ru Loading. C_dL_ Has Been Calculated by Fitting EIS Parameters and Applying Equation 5

	C_dL_ (mF cm^–2^)	α	*R*_ct_ (Ohm)	Active Surface Area (cm^–2^)	Loading (mg cm^–2^)
CF/Ru 0.5	0.27 ± 0.01	0.85	240	4.42	0.02
CF/Ru 0.5 IPA 4%	0.42 ± 0.02	0.77	37	7.1	0.066
CF/Ru 0.5 IPA 6%	0.57 ± 0.02	0.82	30	9.4	0.075
CF/Ru 1	0.12 ± 0.01	0.87	180	1.92	0.025
CF/Ru 1 IPA 5%	2.1 ± 0.1	0.85	12	35	0.04
DOUBLE	15.2 ± 0.6	0.83	8	253	0.06
CF	0.043 ± 0.003	0.91	1700	50 (from BET for 1 cm^2^ electrode)	0

The values of the parameter α are in the range 0.77–0.87, in agreement with values reported for PtRu deposited in cavity microelectrodes.^44^ Assuming a specific capacitance of 60 μF cm^–2^ as reported in ref.^45^, we have also evaluated the active surface area. As a reference, the CF surface area evaluated from the BET area (ref.^29^) has been reported for a 1 cm^2^ CF electrode.

**Figure 8:**
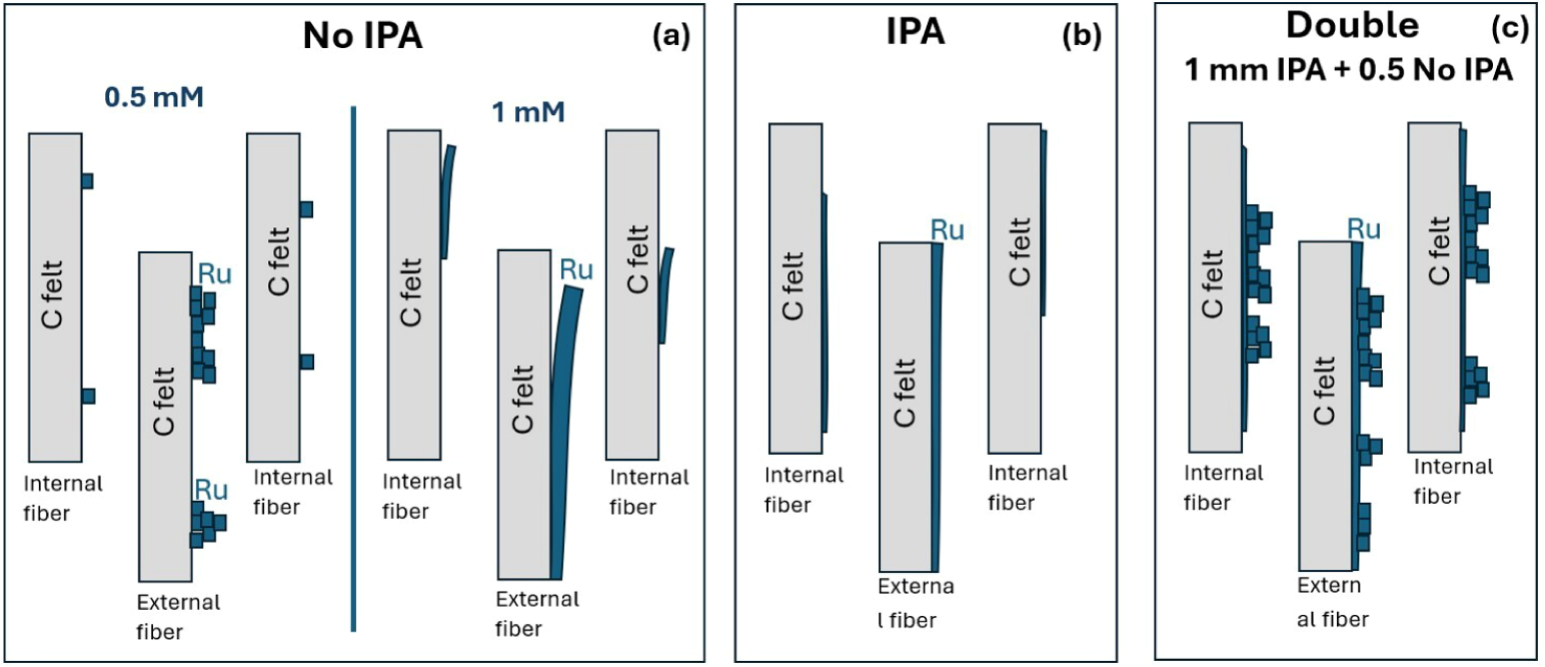
Schematic of the deposition process without or with IPA addition and with different RuCl_3_ concentrations.

The comparison between electrochemical data and the morphology offers some insights into the electrode structure and the effect of IPA addition in the electrodeposition solution. The charge transfer resistance is dependent on the contact area between the supporting carbon felt and the Ru catalyst, while the active surface area is proportional to the number of active sites available for the hydrogen evolution reaction. Structured catalysts with high surface-to-volume ratios are therefore expected to exhibit higher capacitance; lower resistance is expected when uniform coverage of the carbon fibers is achieved. In the case of deposition without IPA addition, the SEM micrograph in Figure 1 clearly shows that deposition with 0.5 mM RuCl_3_ gives rise to structured islands, with poor Ru coverage but with a high surface-to-volume ratio; for 1 mM, an almost continuous Ru film is achieved (at least on the external fibers), even without IPA. According to this observation, a slightly lower *R*_ct_ is obtained for 1 mM, while a higher capacitance is achieved with 0.5 mM no IPA deposition. If we compare the active surface area of CF/Ru electrodes to the CF BET surface area (about 50 cm^2^ for a 1 cm^2^ electrode), values corresponding to less than 10% are obtained for Ru electrodeposited without IPA, again suggesting that the fibers are not completely covered. A schematic picture of the deposition process without IPA is shown in Figure 8a.

The addition of IPA increases the wettability of the CF. As a consequence, the deposition becomes more uniform and also involves the inner carbon fibers, as sketched in Figure 8b. Therefore, higher area coverage is obtained, as confirmed by the lowering of the *R*_ct_ and the increase in the active area. In particular, for CF/Ru 1 mM IPA, the active surface area is close to the available area, suggesting an almost complete coverage of the carbon fibers. Finally, the electrode produced with the double-step procedure combines the complete coverage obtained through the deposition with 1 mM RuCl_3_ and IPA in the first step, with the porous and rough surface achievable through deposition without IPA, as shown in the schematic of Figure 8c. This observation is confirmed by the electrochemical data, clearly indicating an active surface area larger than the available CF area.

The comparison with the literature data in Table 3 shows that the uniform coverage plays a significant role in the electrochemical performance of the electrode. The CF/Ru double layer indeed exhibits a Tafel slope and an overpotential value comparable to those presented in the literature for other Ru-based catalysts operating in acidic environments. However, in the present work, the Ru amount is much lower—only 0.06 mg cm^–2^—thanks to the improved coverage and optimized active area. A more comprehensive comparison with Ru catalysts operating in different electrolytes is shown in Table SI2. Changing from an alkaline (KOH) to an acidic environment usually increases the overpotential and the Tafel slope. The performance further decreases when considering a neutral electrolyte. This was confirmed by testing in a neutral electrolyte (0.1 M Na_2_SO_4_) in order to compare it with the acidic environment (see the linear sweep voltammetry curve in Figure SI6).

**Table 3:** Comparison with Other Ru-Based Catalysts Reported in the Literature

Catalyst	Electrolyte	η (mV) @10 mA cm^–2^	T_slope_ (mV dec^–1^)	Loading	Ref.
CF/Ru Double	0.1 M H_2_SO_4_	67	50	0.06 mg cm^–2^	This work
Ni@Ni_2_ P–Ru	0.5 M H_2_SO_4_	51	35	-	(19)
Ru/NG-750	0.5 M H_2_SO_4_	53	44	-	(20)
Ru/MoS_2_/CP	0.5 M H_2_SO_4_	96	-	-	(21)
Pd–Ru	0.5 M H_2_SO_4_	26	28	-	(22)
Ru/GLC	0.5 M H_2_SO_4_	35	46	0.400 mg cm^–2^	(23)
Ru_1.0_/NF	0.5 M H_2_SO_4_	47	60	∼1.1 wt %	(26)
Ru^0^/CeO_2_	0.5 M H_2_SO_4_	47	41	0.197 mg cm^–2^ or 1.86 wt % Ru	(20)
Ru-MoO_2_	0.5 M H_2_SO_4_	55	44	0.57 mg cm^–2^	(46)

### 3.3 Chemical State and Stability of Best-Performing Electrode

**Figure 9:**
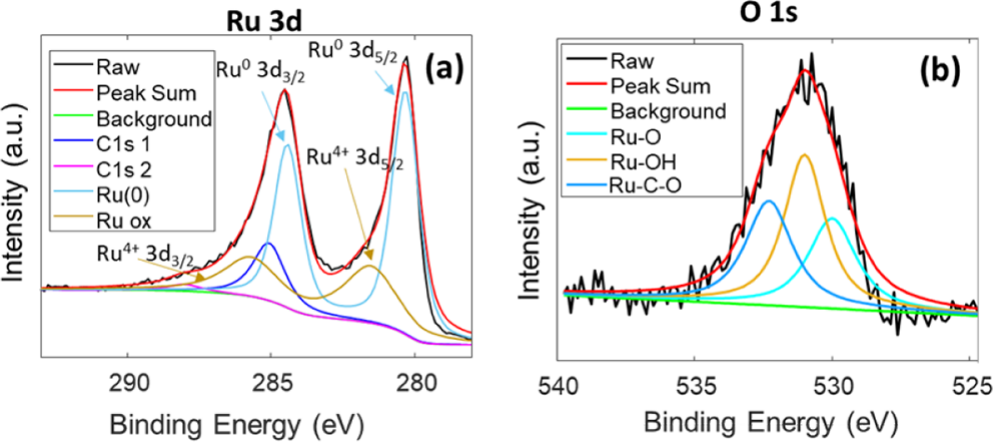
XPS spectra of (a) the 3d Ru peak and (b) the 1s O peak, acquired for the as-prepared CF/Ru electrode prepared by double-step deposition.

To investigate more deeply the CF/Ru double electrode, we also analyzed the chemical composition and chemical states using XPS. Figure 9 shows the XPS spectra acquired in the region of the Ru 3d peak, which includes two spin–orbit peaks assigned to metallic Ru (Ru^0^): Ru 3d_5/2_ (with a BE of 280.5 eV) and Ru 3d_3/2_ (BE of 284.6 eV). Additionally, two peaks at 281.7 and 285.9 eV can be ascribed to some oxidized Ru,^47^ Ru^4+^. This observation is complemented by the analysis of the oxygen 1s region, shown in Figure 9 b. In the fresh electrode, three contributions are visible: at 529.4 eV, characteristic of Ru–O species; at 531 eV, due to the presence of hydroxide surface bonds; and at 532.3 eV, related to the presence of Ru–C–O interactions.^48^ Therefore, we can conclude that the as-prepared catalyst is composed of Ru, covered by a thin Ru oxide layer.

**Figure 10:**
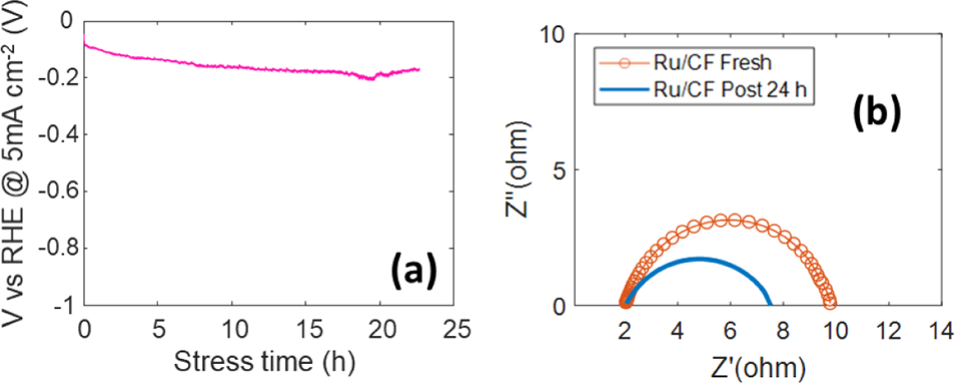
(a) Chronopotentiometry curve under a constant current of −5 mA cm^–2^. (b) EIS measurement performed in the as-prepared fresh electrode and after chronopotentiometry for 24 h.

In order to verify the durability of the best-performing electrode, chronopotentiometry measurements as a function of time were performed up to 24 h, as reported in Figure 10a. For this measurement, the current was kept fixed at −5 mA cm^–2^. No relevant variation in the potential vs RHE required to maintain the current was observed, suggesting that no degradation occurred. Figure 10b shows the comparison between EIS acquired in the as-prepared electrode and after 24 h of operation. After stress, the charge transfer resistance decreases to 5.5 Ohms, while the *C*_dL_ undergoes a slight increase and reaches 16.6 mF cm^–2^.

**Figure 11:**
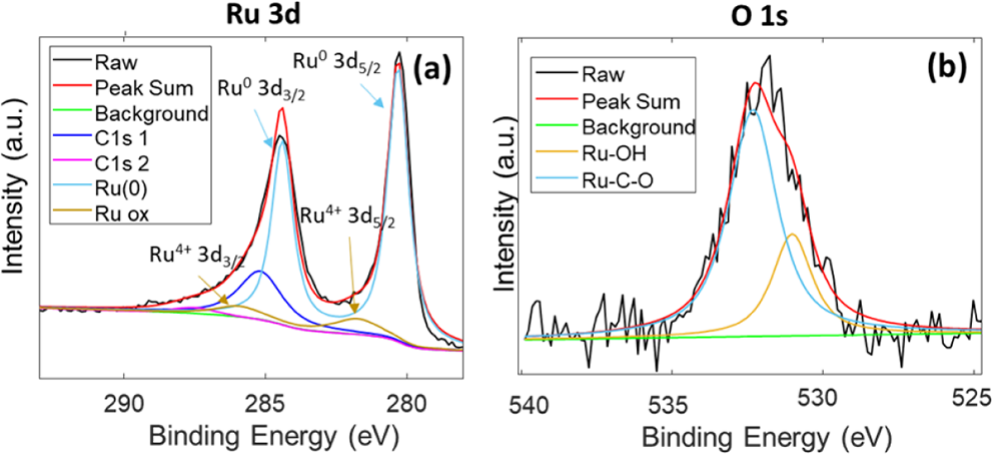
XPS spectra of the 3d Ru peak (a) and (b) 1s O, after chronopotentiometry at −5 mA cm^–2^ in 0.1 M H_2_SO_4_ for 24 h.

The XPS analysis performed after 24 h of operation at −5 mA cm^–2^ is shown in Figure 11. To verify modifications of the chemical state, we have considered the ratio between Ru^0^ and Ru^4+^. Initially, it amounts to 66.6%, while after the HER for 24 h, it increases to 89%, indicating that the operation of Ru in a cathodic region results in its reduction to the metallic state. The same chemical environment can be deduced from the comparison of the Ru 3p peak before and after the chronopotentiometry, as reported in Figure SI 7(a,b). The oxygen peak after chronoamperometry can be fitted by considering the contribution of only two peaks, related to hydroxide surface bonds and Ru–C–O, at 531 and 532.3 eV, respectively. Therefore, afterchronopotentiometry for 24 h, the peak of the Ru–O peak at 529.4 eV completely disappears, confirming the change in the chemical state of Ru at the surface from Ru^4+^ to Ru^0^. Accordingly, lower *R*_ct_ was measured by EIS in the electrode after stress.

To further evaluate the effect of long-term operation, the morphology of the electrode CF/Ru double has been analyzed by SEM after 30 h of operation. As shown in Figure SI8, in the stressed electrode, the Ru film almost completely covers the carbon fibers and exhibits a rough surface, similar to that of the as-prepared electrode. Moreover, in the fresh electrode, some fractures were observed, which, after stress, appear more pronounced in both number and size, although they do not affect the electrochemical performance. This could be due to the reduction of the surface catalyst from Ru oxide to metallic Ru, as observed by XPS and confirmed by EIS, with a consequent loss of oxygen atoms and a volume decrease.

The activity of the CF/Ru double electrode has also been tested in 0.1 M Na_2_SO_4_ at higher current. The chronopotentiometry at −5 mA cm^–2^ and −10 mA cm^–2^ in Na_2_SO_4_ is shown in Figure SI 9. In a neutral environment, the voltage required to maintain −5 mA cm^–2^ increases from 100 mV, obtained in H_2_SO_4_, to 350 mV vs RHE.

## 4 Conclusions

In this work, we adopted a solution-based technique to obtain Ru catalysts for operation in an acidic environment. The catalyst was prepared on carbon felt substrates using electrodeposition, with the aim of achieving the most suitable porous support for operation at a low pH value. The morphology and electrocatalytic properties of the Ru catalyst supported on carbon felt were investigated as a function of RuCl_3_ concentration and upon the addition of a surfactant (IPA) in the deposition solution. The introduction of IPA during Ru electrodeposition (i) increases the wettability of carbon felt, facilitating the coverage of the carbon fibers even in the inner regions, (ii) significantly improves nucleation uniformity, giving rise to smooth Ru layers, and (iii) decreases the competing HER occurring during the electrodeposition. As a consequence, Ru-loaded electrodes electrodeposited with IPA addition exhibit enhanced electrochemical performance, with better charge transfer properties and increased active surface area. To optimize the electrochemical performance, a double-step process was developed, consisting of one deposition with IPA followed by a second deposition without IPA. This approach allows for the combination of the almost complete coverage of the carbon fibers with a smooth Ru layer, achievable via electrodeposition with IPA, with the enhanced surface roughness promoted by electrodeposition without IPA. In this way, it has been shown that it is possible to maximize the number of active catalytic sites while maintaining uniform coverage and Ru loading below 0.1 mg cm^–2^. The proposed method is effective in obtaining an electrode with overpotential and Tafel slope comparable to that of Pt, suitable for operation in an acidic environment, and the electrode stability has been proven for up to 24 h.

## Data Availability

Data will be made available on request

## Supporting Information Available

RuCl_3_ calibration with UV–vis spectroscopy and calculation of Ru loading on CF/Ru; chronopotentiometry acquired during Ru electrodeposition at a constant current of −1.5 mA cm^–2^ for 1000 s without IPA or with IPA added to the RuCl_3_ solution; SEM analyses at a high magnification; EDS analyses for CF/Ru 1 mM with IPA; linear sweep voltammetry (LSV) acquired for CF/Ru electrodes prepared by electrodeposition in 1 mM RuCl_3_ by changing the electrodeposition time; table for comparison with other Ru-based catalysts present in the literature; comparison between linear sweep voltammetry curves acquired in acidic and neutral environments; XPS analyses of CF/Ru double electrode in the Ru 3p region before and after chronopotentiometry at −5 mA cm^–2^ for 24 h; SEM micrographs of the electrode CR/Ru double after 30 h of operation; comparison between chronopotentiometry acquired for different current values and electrolytes (PDF)

## References

[ref1] Bernt M.; Hartig-Weiß A.; Tovini M. F.; El-Sayed H. A.; Schramm C.; Schröter J.; Gebauer C.; Gasteiger H. A. Current Challenges in Catalyst Development for PEM Water Electrolyzers. Chem. Ing. Technol. 2020, 92 (1–2), 31–39. 10.1002/cite.201900101.

[ref2] Carmo M.; Fritz D. L.; Mergel J.; Stolten D. A. A comprehensive review on PEM water electrolysis. Int. J. Hydrogen Energy. 2013, 38 (12), 4901–4934. 10.1016/j.ijhydene.2013.01.151.

[ref3] Yang H.; Zhang Y.; Hu F.; Wang Q. Urchin-like CoP nanocrystals as hydrogen evolution reaction and oxygen reduction reaction dual-electrocatalyst with superior stability. Nano Lett. 2015, 15 (11), 7616–7620. 10.1021/acs.nanolett.5b03446. 26474359

[ref4] Zhang Y.; Gong Q.; Li L.; Yang H.; Li Y.; Wang Q. MoSe_2_ porous microspheres comprising monolayer flakes with high electrocatalytic activity. Nano Res. 2015, 8 (4), 1108–1115. 10.1007/s12274-014-0590-0.

[ref5] Kibsgaard J.; Jaramillo T. F. Molybdenum Phosphosulfide: An Active, Acid-Stable, Earth-Abundant Catalyst for the Hydrogen Evolution Reaction Angew. Chem., Int. Ed. 2014, 53, 14433–14437. 10.1002/anie.201408222. 25359678

[ref6] Buhler M.; Holzapfel P.; McLaughlin D.; Thiele S. From catalyst coated membranes to porous transport electrode based configurations in PEM water electrolyzers. J. Electrochem. Soc. 2019, 166 (14), F1070 10.1149/2.0581914jes.

[ref7] Buhler M.; Hegge F.; Holzapfel P.; Bierling M.; Suermann M.; Vierrath S.; Thiele S. Optimization of anodic porous transport electrodes for proton exchange membrane water electrolyzers. J. Mater. Chem. 2019, 7 (47), 26984–26995. 10.1039/C9TA08396K.

[ref8] Fleming G. J.; Fleming P. J. Development and optimization of porous carbon papers suitable for gas diffusion electrodes. 2001.

[ref9] Kus P.; Ostroverkh A.; Sevcikova K.; Khalakhan I.; Fiala R.; Skala T.; Tsud N.; Matolin V. Magnetron sputtered Ir thin film on TiC-based support sublayer as low-loading anode catalyst for proton exchange membrane water electrolysis. Int. J. Hydrogen Energy 2016, 41 (34), 15124–15132. 10.1016/j.ijhydene.2016.06.248.

[ref10] Laube A.; Hofer A.; Ressel S.; Chica A.; Bachmann J.; Struckmann T. PEM water electrolysis cells with catalyst coating by atomic layer deposition. Int. J. Hydrogen Energy 2021, 46 (79), 38972–38982. 10.1016/j.ijhydene.2021.09.153.

[ref11] Bachmann J. Atomic layer deposition, a unique method for the preparation of energy conversion devices Beilstein. J. Nanotechnol. 2014, 5, 245–248. 10.3762/bjnano.5.26. PMC3999862 24778945

[ref12] Lee W.- J.; Bera S.; Kim C. M.; Koh E.- K.; Hong W.- P.; Oh S.- J.; Cho E.; Kwon S.- H. Synthesis of highly dispersed Pt nanoparticles into carbon supports by fluidized bed reactor atomic layer deposition to boost PEMFC performance. NPG Asia Mater. 2020, 12 (1), 40 10.1038/s41427-020-0223-x.

[ref13] Lu X.; Zhao C. Electrodeposition of hierarchically structured three-dimensional nickel–iron electrodes for efficient oxygen evolution at high current densities. Nat. Commun. 2015, 6, 6616 10.1038/ncomms7616. 25776015 PMC4382694

[ref14] Leofanti G.; Padovan M.; Tozzola G.; Venturelli B. Surface area and pore texture of catalysts. CatalToday 1998, 41, 207–219. 10.1016/S0920-5861(98)00050-9.

[ref15] Eiler K.; Suriñach S.; Sort J.; Pellicer E. Mesoporous Ni-rich Ni–Pt thin films: Electrodeposition, characterization and performance toward hydrogen evolution reaction in acidic media. Appl. Catal., B 2020, 265, 118597 10.1016/j.apcatb.2020.118597.

[ref16] Zhao Z.; Yang H.; Li M.; Gudi C.; Kanumuru K. V.; Voigt R.; Liu O. B. T.; Chen Y.; Sun H. Engineering heterogeneous domains and interfaces in shape memory fibers for tunable responsive behaviors. Chem. Eng. J. 2024, 480, 147936 10.1016/j.cej.2023.147936.

[ref17] Zhu J.; Cai L.; Tu Y.; Zhang L.; Zhang W. Emerging ruthenium single-atom catalysts for the electrocatalytic hydrogen evolution reaction. J. Mater. Chem. A 2022, 10, 15370–15389. 10.1039/D2TA03860A.

[ref18] Wang J.; Wei Z.; Mao S.; Li H.; Wang Y. Highly uniform Ru nanoparticles over N-doped carbon: pH and temperature-universal hydrogen release from water reduction. Energy Environ. Sci. 2018, 11, 800–806. 10.1039/C7EE03345A.

[ref19] Liu Y.; Liu S.; Wang Y.; Zhang Q.; Gu L.; Zhao S.; Xu D.; Li Y.; Bao J.; Dai Z. Ru Modulation Effects in the Synthesis of Unique Rod-like Ni@Ni 2 P–Ru Heterostructures and Their Remarkable Electrocatalytic Hydrogen Evolution Performance. Am. Chem. Soc. 2018, 140, 2731–2734. 10.1021/jacs.7b12615. 29415541

[ref20] Demir E.; Akbayrak S.; Onal A. M.; Ozkar S. High Performance Electrocatalytic Reaction of Hydrogen and Oxygen on Ruthenium Nanoclusters. ACS Appl. Mater. Interfaces 2018, 10, 6299–6308. 10.1021/acsami.7b17469. 29420007

[ref21] Liu J.; Zheng Y.; Zhu D.; Vasileff A.; Ling T.; Qiao S.- Z. Identification of pH-dependent synergy on Ru/MoS2 interface: A comparison of alkaline and acidic hydrogen evolution. Nanoscale 2017, 9, 16616–16621. 10.1039/C7NR06111K. 29075731

[ref22] Liu S.; Zhang Q.; Bao J.; Li Y.; Dai Z.; Gu L. Significantly Enhanced Hydrogen Evolution Activity of Freestanding Pd-Ru Distorted Icosahedral Clusters with less than 600 Atoms. Chem. Eur. J. 2017, 23, 18203–18207. 10.1002/chem.201702913. 28741317

[ref23] Chen Z.; Lu J.; Ai Y.; Ji Y.; Adschiri T.; Wan L. Ruthenium/Graphene-like Layered Carbon Composite as an Efficient Hydrogen Evolution Reaction Electrocatalyst. ACS Appl. Mater. Interfaces 2016, 8, 35132 10.1021/acsami.6b09331. 27966849

[ref24] Wang J.; Wei Z.; Mao S.; Li H.; Wang Y. Highly uniform Ru nanoparticles over N-doped carbon: pH and temperature-universal hydrogen release from water reduction. Energy Environ. Sci. 2018, 11, 800–806. 10.1039/C7EE03345A.

[ref25] Kibsgaard J.; Hellstern T. R.; Choi S. J.; Reinecke B. N.; Jaramillo T. F. Mesoporous Ruthenium/Ruthenium Oxide Thin Films: Active Electrocatalysts for the Oxygen Evolution Reaction. ChemElectrochem 2017, 4, 2480 10.1002/celc.201700334.

[ref26] Xia J.; Volokh M.; Peng G.; Fu Y.; Wang X.; Shalom M. Low-Cost Porous Ruthenium Layer Deposited on Nickel Foam as a Highly Active Universal-pH Electrocatalyst for the Hydrogen Evolution Reaction. ChemSuschem 2019, 12, 2780 10.1002/cssc.201900472. 30938925

[ref27] Zangari G.; Villa M. Electrochemical Behavior of Nickel in Acidic Sulfate Electrolytes. ECS Trans 2008, 6, 297 10.1149/1.2943250.

[ref28] Liu B.; Simonsen S. B.; Hjelm J. Morphological properties and electrochemical performance for compressed carbon-fiber electrodes in Redox Flow Batteries. J. Electrochem. Soc. 2024, 171, 040503 10.1149/1945-7111/ad36e5.

[ref29] Bertin E.; Garbarino S.; Guay D.; Solla-Gullón J.; Vidal-Iglesias F. J.; Feliu J. M. Electrodeposited platinum thin films with preferential (100) orientation: Characterization and electrocatalytic properties for ammonia and formic acid oxidation. J. Power Sources 2013, 225, 323–329. 10.1016/j.jpowsour.2012.09.090.

[ref30] Bi S.; Hao S.; Lia L.; Zhang S. Bio-bar-code dendrimer-like DNA as signal amplifier for cancerous cells assay using ruthenium nanoparticle-based ultrasensitive chemiluminescence detection. Chem.Commun. 2010, 46, 6093–6095. 10.1039/C0CC01409E. 20652188

[ref31] Tåg C.-M.; Toiviainen M.; Juuti M.; Rosenholm J. B.; Backfolk K.; Gane P. A. C. The Effect of Isopropyl Alcohol and Non-Ionic Surfactant Mixtures on the Wetting of Porous Coated Paper. Transp Porous Med. 2012, 94, 225–242. 10.1007/s11242-012-0001-5.

[ref32] Ou W.; Zhang Y.; Li H. Effects of IPA on texturing process for mono-crystalline silicon solar cell in TMAH solution. Mater. Sci. Forum. 2011, 685, 31–37. 10.4028/www.scientific.net/MSF.685.31.

[ref33] Zubel I.; Kramkowska M. The effect of isopropyl alcohol on etching rate and roughness of (1 0 0) Si surface etched in KOH and TMAH solutions. Sens. Actuators, A 2001, 93, 138–147. 10.1016/S0924-4247(01)00648-3.

[ref34] Singh P. K.; Kumar R.; Lal M.; Singh S. N.; Das B. K. Effectiveness of anisotropic etching of silicon in aqueous alkaline solutions. Sol. Energy Mater. Sol. Cells 2001, 70, 103–113. 10.1016/S0927-0248(00)00414-1.

[ref35] Ju M.; Balaji N.; Park C.; Nguyen H.T.T.; Cui J.; Oh D.; Jeon M.; Kang J.; Shim G.; Yi J. The effect of small pyramid texturing on the enhanced passivation and efficiency of single c-Si solar cells. RSC Adv. 2016, 6, 49831–49838. 10.1039/C6RA05321A.

[ref36] Abdur-Rahman E.; Alghoraibi I.; Alkurdi H. Effect of Isopropyl Alcohol Concentration and Etching Time on Wet Chemical Anisotropic Etching of Low-Resistivity Crystalline Silicon Wafer. Int. J. Anal Chem. 2017, 2017, 7542870 10.1155/2017/7542870. 28831284 PMC5554996

[ref37] Bauer A.; Gyenge E. L.; Oloman C. W. Electrodeposition of Pt–Ru nanoparticles on fibrous carbon substrates in the presence of nonionic surfactant: Application for methanol oxidation. Electrochim. Acta 2006, 51, 5356–5364. 10.1016/j.electacta.2006.02.006.

[ref38] Liu Y.; Wu Z.; Qin Z.; Liu Y.; Hu W. Recent progress in inhibition of hydrogen evolution reaction in alkaline Al-air batteries. Natl. Sci. Open. 2024, 3, 20240037 10.1360/nso/20240037.

[ref39] Harrington D. A.; Conway B. E. AC Impedance of Faradaic reactions involving electrosorbed intermediates—I Kinetic theory. Electrochim. Acta 1987, 32, 1703–1712. 10.1016/0013-4686(87)80005-1.

[ref40] Krstajić N.; Popović M.; Grgur B.; Vojnović M.; Šepa D. On the kinetics of the hydrogen evolution reaction on nickel in alkaline solution Part The mechanism. J. Electroanal.Chem 2001, 512, 16–26. 10.1016/S0022-0728(01)00590-3.

[ref41] Tian X.; Zhao P.; Sheng W. Hydrogen Evolution and Oxidation: Mechanistic Studies and Material Advances. Adv. Mater. 2019, 31, 1808066 10.1002/adma.201808066. 30932265

[ref42] Khadke P.; Tichter T.; Boettcher T.; Muench F.; Ensinger W.; Roth C. A simple and effective method for the accurate extraction of kinetic parameters using differential Tafel plots. Sci. Rep 2021, 11, 8974 10.1038/s41598-021-87951-z. 33903627 PMC8076256

[ref43] Mateos M.; Meunier-Prest R.; Suisse J. M.; Bouvet M. Modulation of the organic heterojunction behavior, from electrografting to enhanced sensing properties. Sensord Actuoators B. Chemical 2019, 229, 126968 10.1016/j.snb.2019.126968.

[ref44] Tremblay M.-L.; Martin M. H.; Lebouin C.; Lasia A.; Guay D. Determination of the real surface area of powdered materials in cavity microelectrodes by electrochemical impedance spectroscopy. Electrochim. Acta 2010, 55, 6283–6291. 10.1016/j.electacta.2009.11.006.

[ref45] Jiang Y.; Yang R.; Shi G.; Xia J.; Chen J.; Su Q.; Chen Pt-like electrocatalytic behavior of Ru–MoO2 nanocomposites for the hydrogen evolution reaction. J. Mater. Chem. A 2017, 5, 5475–5485. 10.1039/C6TA09994G.

[ref46] Mao X.; Liu Z.; Lin C.; Li J.; Shen P. K. Bimetallic ruthenium-nickel alloy nanostructure supported on nickel foam for efficient alkaline hydrogen evolution at large current density Inorg. Chem. Front. 2023, 10, 558–566. 10.1039/D2QI02084J.

[ref47] Morgan D. J. Resolving ruthenium: XPS studies of common ruthenium materials. Surf. Interface Anal. 2015, 47, 1072–1079. 10.1002/sia.5852.

[ref48] Jiang Y.; Yang R.; Shi G.; Xia J.; Chen J.; Su Q. C. Pt-like electrocatalytic behavior of Ru–MoO2 nanocomposites for the hydrogen evolution reaction. J. Mater. Chem. A 2017, 5, 5475–5485. 10.1039/C6TA09994G.

