# Supplementary material for: Efficient Electrochemical Methods for Low-Loading Ru Deposition on Carbon Electrodes as a Hydrogen Evolution Reaction Catalyst in an Acidic Environment
Source: ACS Appl Energy Mater. 2025 Apr 29;8(9):5698–707. doi: 10.1021/acsaem.4c03349 (PMC12077262; doi:10.1021/acsaem.4c03349)
Supplement: Supplementary file 1 — ae4c03349_si_001.pdf [file ae4c03349_si_001.pdf]

Supporting Information

Efficient electrochemical methods for low loading Ru deposition on carbon electrodes as hydrogen evolution reaction catalyst in acidic environment

Rachela Gabriella Milazzo [a] , Nino Marino [a], Giuseppe Tranchida [a,b] , Corrado Bongiorno [a], Luca Pulvirenti [b], Letizia Fusto[a], Guido Gugliemo Condorelli[b], Salvatore Antonino Lombardo[a] , Stefania Maria Serena Privitera [a].*

[a] CNR-IMM VIII Strada 5, 95121 ZI Catania (CT) Italy;

[b] Department of Chemical Sciences, University of Catania, Viale Andrea Doria, 6, 95125 Catania, Italy

**Corresponding Author e-mail:* stefania.privitera@cnr.it

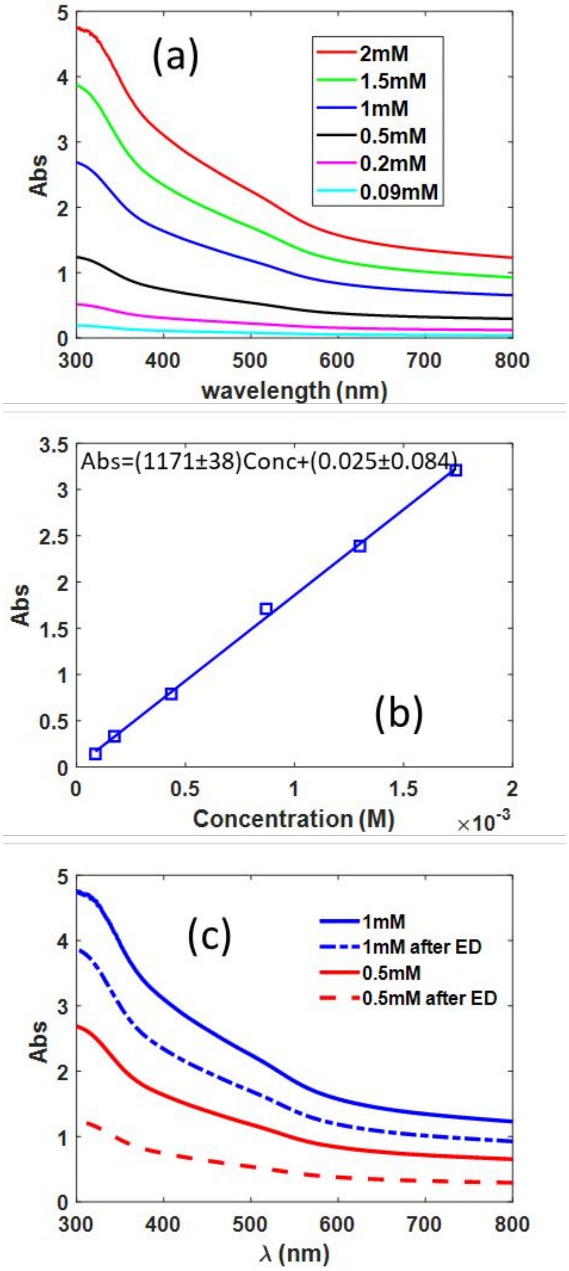

Figure SI 1: $RuCl_3$ calibration with UV Vis spectroscopy and calculation of Ru loading on CF/Ru electrodes. (a) Absorbance at various solution concentration, (b) the calibration curve with equation Abs=(1171 ± 38)Conc + (0.025 ± 0.0084) and (c) the absorbance before (solid lines) and after electro-deposition (dashed lines).

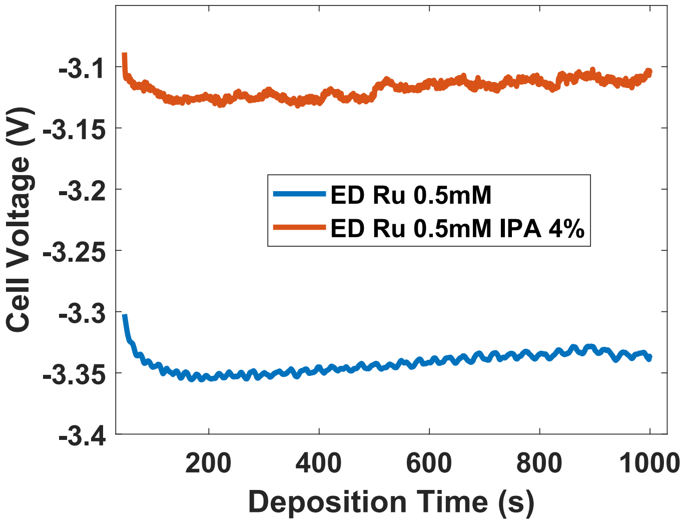

Figure SI 2: Chronopotentiometry acquired during Ru electrodeposition at constant current of -1.5 mA cm^{-2} for 1000 s without IPA or with IPA added in the $RuCl_3$ solution.

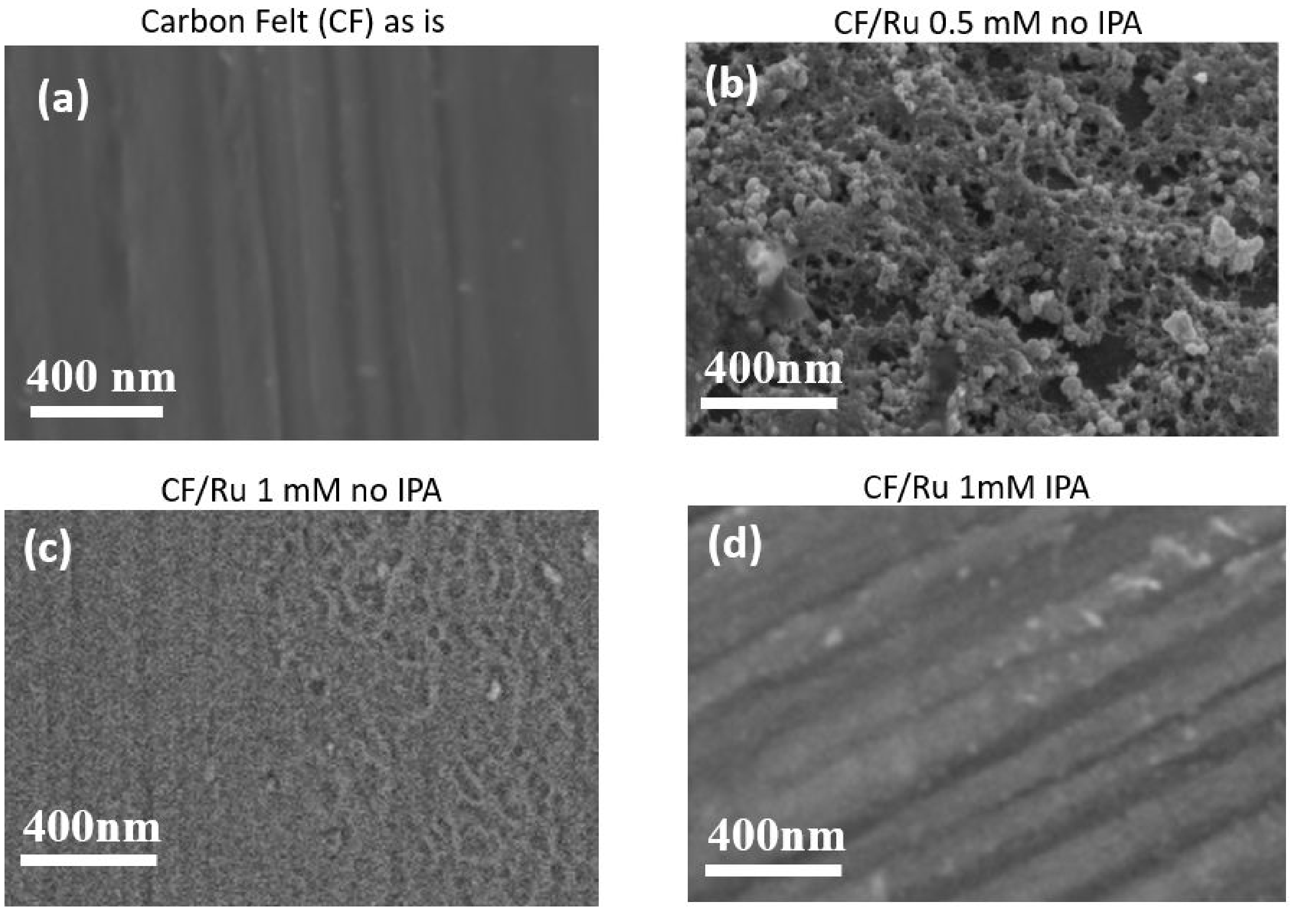

Figure SI 3: SEM analyses at high magnification, showing the effect of concentration and IPA addition on the morphology. (a) Carbon felt (CF) as is, before Ru electrodeposition; (b) CF after Ru electrodeposition without IPA with 0.5 mM $RuCl_3$, giving rise to very rough and porous structures. (c) CF after deposition without IPA in 1 mM $RuCl_3$, exhibiting a smoother but porous Ru layer. (d) CF after deposition with IPA and 1 mM $RuCl_3$

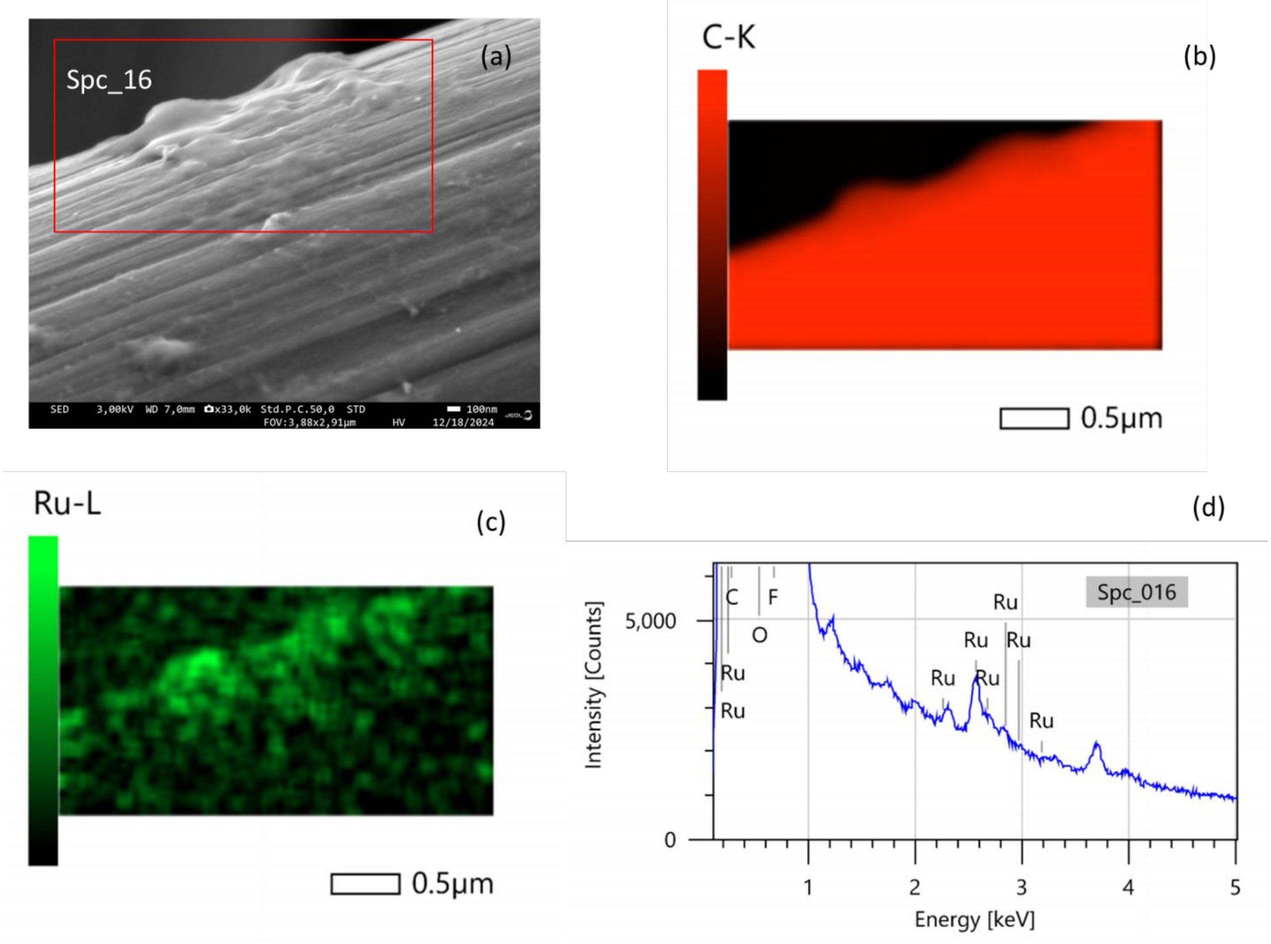

Figure SI 4: SEM and EDS analyses for CF/Ru 1mM with IPA, showing the presence of a very thin and smooth Ru layer covering the Carbon fiber. The computed atomic concentrations are reported in the Table 1

Table SI 1: Atomic concentration computed from EDS of Figure SI 3 (a).

Element	C	O	F	Ru
Atomic %	69.86	21.31	8.71	0.12

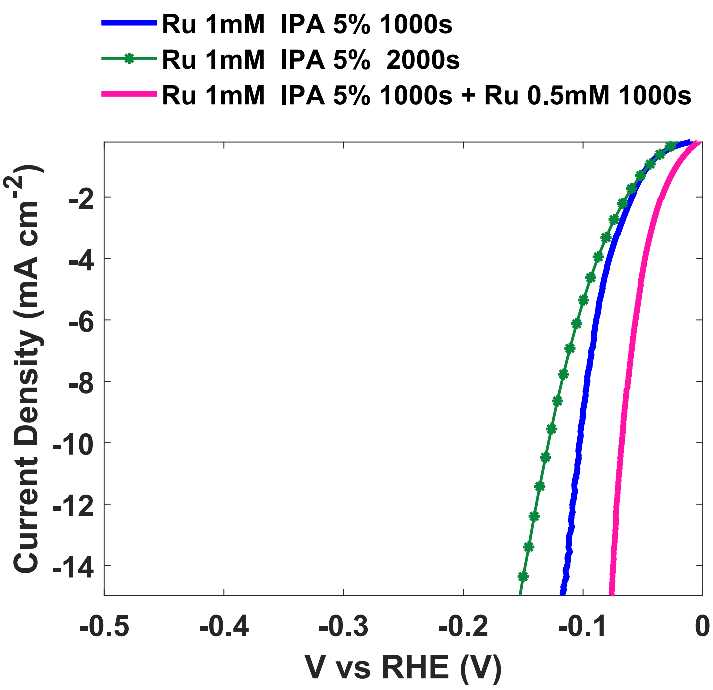

Figure SI 5: Linear sweep voltammetry (LSV) acquired for CF/Ru electrodes prepared by electrodeposition in 1mM $RuCl_3$. Comparison between the electrode electrodeposited with IPA addition for 1000 s, for 2000s and for 1000s followed by a second deposition in 0.5 mM $RuCl_3$ for 1000s without IPA.

Table SI 2

Catalyst	Electrolyte	η (mV) @10mA cm^{-2}	T_{slope}(mV dec^{-1})	Loading	Ref.
**CF/Ru Double**	**0.1 M H_2SO_4**	**67**	**50**	**0.06 mg cm^{-2}**	**This work**
Ru NPs@CN	1 M KOH	32	53	3.18 wt%	[1]
Ni@Ni_2 P−Ru	0.5 M H_2SO_4	51	35	-	[2]
Ni@Ni_2 P−Ru	1 M KOH	31	41	-	[2]
Ru/NG-750	1 M KOH	8	30	-	[3]
Ru/NG-750	0.5 M H_2SO_4	53	44	-	[3]
Ru/MoS_2/CP	1 M KOH	13	60		[4]
Ru/MoS_2/CP	0.5 M H_2SO_4	96	-	-	[4]
Pd-Ru	0.5 M H_2SO_4	26	28	-	[5]
Ru/GLC	0.5 M H_2SO_4	35	46	0.400 mg cm^{-2}	[6]
$Ru_{1.0}$/NF	1 M KOH	22	33	~1.1 wt%	[7]
$Ru_{1.0}$/NF	0.5 M H_2SO_4	47	60	~1.1 wt%	[7]
$Ru_{1.0}$/NF	1 M PBS	52	63	~1.1 wt%	[7]
Ru^0/CeO_2	0.5 M H_2SO_4	47	41	0.197 mg cm^{-2} or 1.86 wt% Ru	[8]
RuP_2@NPC	0.5 M H_2SO_4	38	38	23.3 wt%	[9]
RuP_2@NPC	1 M PBS	57	87	23.3 wt%	[9]
RuP_2@NPC	1 M KOH	52	69	23.3 wt%	[9]
Ru@C_2N	0.5 M H_2SO_4	22	30	-	[10]
Ru@C_2N	1 M KOH	17	38	-	[10]
Ru-MoO_2	1 M KOH	29	312	0.285 mg cm^{-2}	[11]
Ru-MoO_2	0.5 M H_2SO_4	55	44	0.57 mg cm^{-2}	[11]
**NF/Ru**	**1 M KOH**	**22**	**35**	**0.06 mg cm^{-2}**	**This work**

1. J. Wang, Z. Wei, S. Mao, H. Li, Y. Wang, Highly uniform Ru nanoparticles over N-doped carbon: pH and temperature-universal hydrogen release from water reduction Energy Environ. Sci., 2018, 11, 800;
2. Y. Liu, S. Liu, Y. Wang, Q. Zhang, L. Gu, S. Zhao, D. Xu, Y. Li, J. Bao, Z. Dai, Ru Modulation Effects in the Synthesis of Unique Rod-like Ni@Ni2P–Ru Heterostructures and Their Remarkable Electrocatalytic Hydrogen Evolution Performance J. Am. Chem. Soc., 2018, 140, 2731;
3. E. Demir, S. Akbayrak, A. M. Onal, S. Ozkar, High Performance Electrocatalytic Reaction of Hydrogen and Oxygen on Ruthenium Nanoclusters ACS Appl. Mater. Interfaces 2018, 10, 6299;
4. J. Liu, Y. Zheng, D. Zhu, A. Vasileff, T. Ling, S. Z. Qiao, Identification of pH-dependent synergy on Ru/MoS2 interface: a comparison of alkaline and acidic hydrogen evolution Nanoscale 2017, 9, 16616;

5. S. Liu, Q. Zhang, J. Bao, Y. Li, Z. Dai, L. Gu, Significantly Enhanced Hydrogen Evolution Activity of Freestanding Pd-Ru Distorted Icosahedral Clusters with less than 600 Atoms Chem. Eur. J. 2017, 23, 18203;
6. Z. Chen, J. Lu, Y. Ai, Y. Ji, T. Adschiri, L. Wan, Ruthenium/Graphene-like Layered Carbon Composite as an Efficient Hydrogen Evolution Reaction Electrocatalyst ACS Appl. Mater. Interfaces 2016, 8, 35132;
7. J. Xia, M. Volokh, G. Peng, Y. Fu, X. Wang, M. Shalom, Low-Cost Porous Ruthenium Layer Deposited on Nickel Foam as a Highly Active Universal-pH Electrocatalyst for the Hydrogen Evolution Reaction ChemSusChem 2019, 12, 2780;
8. E. Demir, S. Akbayrak, A. M. Onal, S. Ozkar, Nanoceria-supported ruthenium(0) nanoparticles: highly active and stable catalysts for hydrogen evolution in water ACS Appl. Mater. Interfaces 2018, 10, 6299-6308;
9. Z. Pu, I. S. Amiinu, Z. Kou, W. Li, S. Mu RuP_2 based catalysts with platinum like activity and higher durability for the hydrogen evolution reaction at all pH values Angew. Chem. 2017, 56, 11559-11564;
10. Mahmood, J., Li, F., Jung, SM. et al. An efficient and pH-universal ruthenium-based catalyst for the hydrogen evolution reaction. Nature Nanotech 12, 441–446 (2017);
11. P. Jiang, Y. Yang, R. Shi, G. Xia, J. Chen, J. Su, Q. Chen, Pt-like electrocatalytic behavior of Ru–MoO2 nanocomposites for the hydrogen evolution reaction J. Mater. Chem. A, 2017,5, 5475-5485.

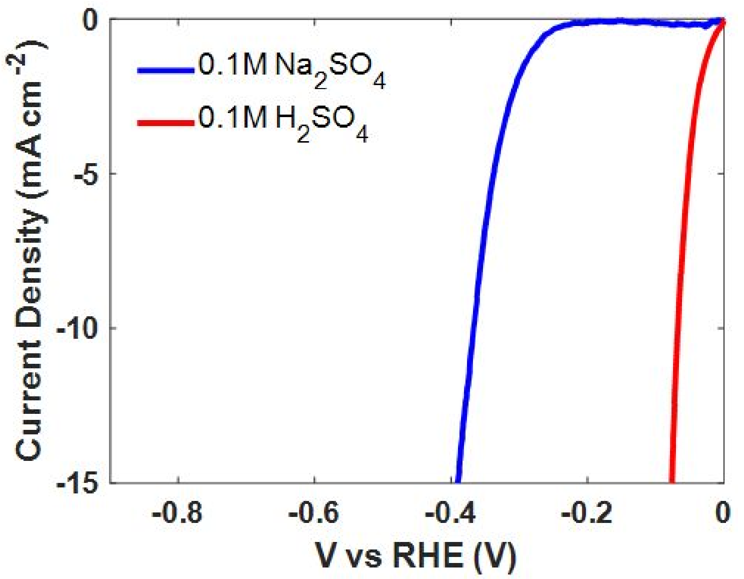

Figure SI 6: Comparison between linear sweep voltammetry curves acquired in acidic and in neutral environment for the electrode produced by the double step deposition.

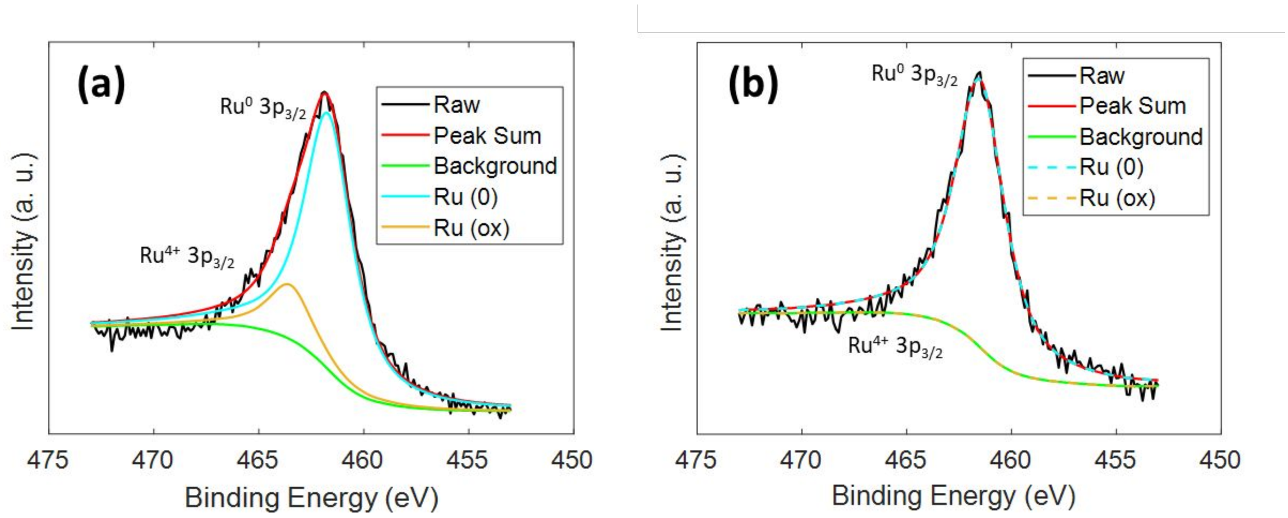

Figure SI 7: XPS analyses of CF/Ru double electrode in the Ru 3p region before (a) and after (b) chronopotentiometry at -5mA cm^{-2} for 24h. The peak at 461.31 eV is due to Ru in the metallic state, that at 463.3 to Ru^{4+}.

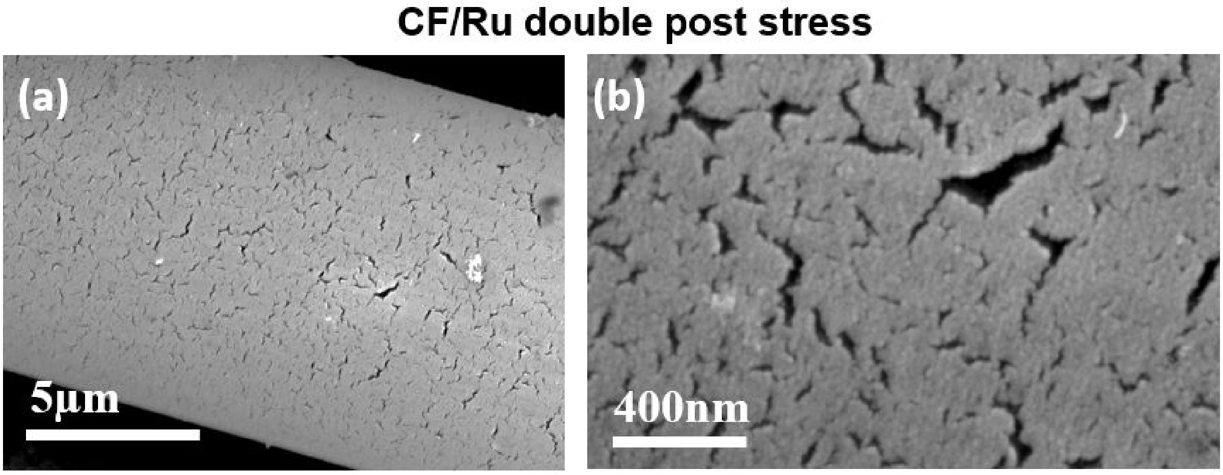

Figure SI 8: SEM micrographs of the electrode CR/Ru double after 30 hours of operation at low (a) and high magnification (b).

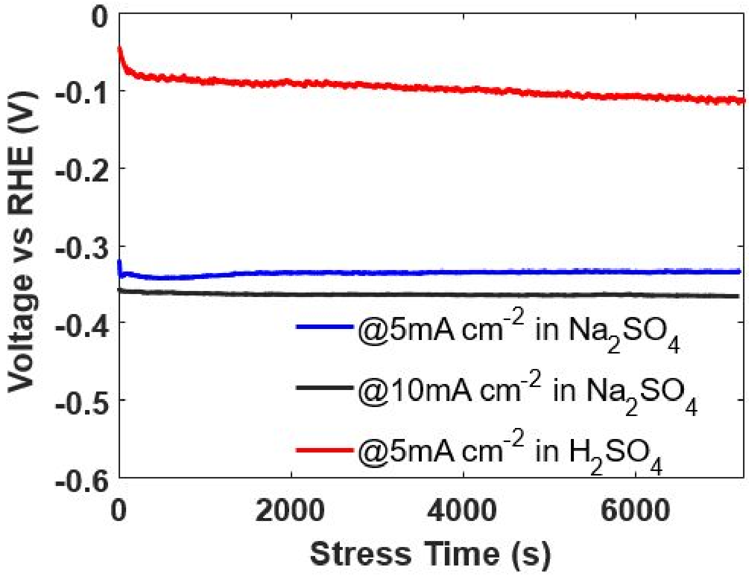

Figure SI 9: Chronopotentiometry acquired during constant current operation: comparison between different current values and electrolytes.
